# Prediction and prevention of preeclampsia in women with preexisting diabetes: the role of home blood pressure, physical activity, and aspirin

**DOI:** 10.3389/fendo.2023.1166884

**Published:** 2023-08-08

**Authors:** Nicoline Callesen Do, Marianne Vestgaard, Sidse Kjærhus Nørgaard, Peter Damm, Elisabeth R. Mathiesen, Lene Ringholm

**Affiliations:** ^1^ Center for Pregnant Women with Diabetes, Rigshospitalet, Copenhagen, Denmark; ^2^ Department of Endocrinology and Metabolism, Rigshospitalet, Copenhagen, Denmark; ^3^ Department of Clinical Medicine, University of Copenhagen, Copenhagen, Denmark; ^4^ Department of Obstetrics, Rigshospitalet, Copenhagen, Denmark

**Keywords:** pregnancy, preexisting diabetes, preeclampsia, hypertension, home blood pressure, physical activity, sedentary behavior, aspirin

## Abstract

Women with type 1 or type 2 (preexisting) diabetes are four times more likely to develop preeclampsia compared with women without diabetes. Preeclampsia affects 9%–20% of pregnant women with type 1 diabetes and 7%–14% of pregnant women with type 2 diabetes. The aim of this narrative review is to investigate the role of blood pressure (BP) monitoring, physical activity, and prophylactic aspirin to reduce the prevalence of preeclampsia and to improve pregnancy outcome in women with preexisting diabetes. Home BP and office BP in early pregnancy are positively associated with development of preeclampsia, and home BP and office BP are comparable for the prediction of preeclampsia in women with preexisting diabetes. However, home BP is lower than office BP, and the difference is greater with increasing office BP. Daily physical activity is recommended during pregnancy, and limiting sedentary behavior may be beneficial to prevent preeclampsia. White coat hypertension in early pregnancy is not a clinically benign condition but is associated with an elevated risk of developing preeclampsia. This renders the current strategy of leaving white coat hypertension untreated debatable. A beneficial preventive effect of initiating low-dose aspirin (150 mg/day) for all in early pregnancy has not been demonstrated in women with preexisting diabetes.

## 1 Introduction

Preeclampsia is a systemic disorder of pregnancy characterized by increased blood pressure (BP) and proteinuria or symptoms of other maternal organ dysfunction (1). Today, preeclampsia is the main cause of maternal and perinatal morbidity and mortality (2, 3) and an important contributor to preterm delivery (4). Worldwide, 3%–5% of all pregnancies are affected by preeclampsia (3, 5, 6). In particular, women with type 1 and type 2 (preexisting) diabetes, hypertension, nephropathy, and/or previous preeclampsia are at high risk of developing preeclampsia (3, 7–11). Control of hypertension with antihypertensive medication reduces the risk of preeclampsia and other adverse maternal and perinatal complications (12–14) with no increase in the risk of small for gestational age infants (14). To date, no curative treatment for preeclampsia is available, and the only cure is delivery of the fetus and of the placenta (2, 3, 6–9, 15).

In women with preexisting diabetes, tight glycemic control prior to and during pregnancy is of utmost importance to reduce the risk of preeclampsia and other adverse pregnancy outcomes (16–19). Diabetes management during pregnancy includes frequent adjustments in insulin doses based on blood glucose monitoring and focuses on adequate diet, physical activity, and gestational weight gain (16, 17, 19). It is also important to screen for proteinuria and to monitor BP and the fetal growth. When indicated, treatment with antihypertensive medication can be initiated or intensified to control BP and urinary albumin excretion (19).

Despite extensive research (16, 20) and improved clinical management of diabetes in pregnancy (21, 22), women with preexisting diabetes are four times more likely to develop preeclampsia compared with women without diabetes (2, 20, 23). Preeclampsia affects 9%–20% of pregnant women with type 1 diabetes (20, 22–24) and 7%–14% of pregnant women with type 2 diabetes (22–25). Given the severity of preeclampsia and the high prevalence in women with preexisting diabetes, prediction, screening, and prevention are crucial yet challenging because of the multifactorial causes of preeclampsia.

Prediction of preeclampsia with BP as an important contributor has been studied in the general pregnant population (3, 26, 27). Reduced physical activity may be associated with development of preeclampsia, and physical activity has been widely studied and seems to have a beneficial effect on the prevention of preeclampsia (28–30). As a pharmacological preventive strategy, prophylactic aspirin is recommended by international societies for women at high risk of preeclampsia (13, 31, 32).

This narrative review investigates the role of BP monitoring, physical activity, and prophylactic aspirin to reduce the prevalence of preeclampsia and to improve pregnancy outcome in women with preexisting diabetes.

## 2 Preeclampsia

The diagnostic criteria for preeclampsia include hypertension in combination with proteinuria or new onset of symptoms of maternal organ dysfunction (thrombocytopenia, impaired liver function, renal insufficiency, pulmonary edema, or cerebral or visual symptoms) (1) (**Table 1**). Fetal growth restriction has also been proposed as a diagnostic criterion in combination with hypertension but without an international consensus regarding this diagnostic criterion (13, 31–34).

**Table 1:** Summary of the 2013 diagnostic criteria for preeclampsia from the American College of Obstetricians and Gynecologists (1).

**Hypertension:** BP ≥ 140 mmHg systolic and/or ≥ 90 mmHg diastolic measured on two occasions at least 4 h apart after 20 gestational weeks.
*and coexistence of*
**Proteinuria:** ≥ 1+ on a urine dipstick of sterile urine
*and/or*
**New onset of symptoms of other organ dysfunction** (one or more of the following):
**Thrombocytopenia:** < 100 × 10^9^/L
**Impaired liver function:** Elevated liver enzymes to twice the normal level
**Renal insufficiency:** Serum creatinine > 100 µmol/L or a doubling of the serum creatinine concentration in the absence of other renal diseases
**Pulmonary edema**
**Cerebral or visual symptoms**

Preeclampsia is often subclassified on the basis of the gestational age at delivery, as maternal and perinatal morbidity varies depending on this timing (26, 35). Preterm preeclampsia with delivery before 37 weeks is, in general, considered more severe than term preeclampsia with delivery after 37 weeks both for the woman and her fetus (26).

### 2.1 Development of preeclampsia

It is still not fully understood why some women develop preeclampsia while others do not. However, development of preeclampsia is, in general, considered to be caused by a combination of abnormal placentation and systemic maternal endothelial dysfunction (3, 7–9, 15).

During normal placentation in early pregnancy, syncytiotrophoblasts invade the uterine spiral arteries, leading to an adaptation and remodeling of the uterine spiral arteries to accommodate the increased blood flow needed to the placenta and fetus (3, 7–9, 15). In pregnancies later complicated by preeclampsia, early invasion of the syncytiotrophoblasts is impaired, with a subsequent deficient remodeling of the uterine spiral arteries. This impaired remodeling leads to narrow placental vessels with a high velocity and a turbulent flow limiting oxygen exchange, which results in placental ischemia and oxidative stress (3, 7–9, 15). The cause of placental dysfunction may be a combination of maternal preexisting risk factors, genetic factors, and immunological factors (3, 7–9, 15). Abnormal placentation may especially be part of the pathogenesis when preeclampsia develops early (6).

Abnormal placentation leads to release of antiangiogenic factors and inflammatory cytokines into the maternal systemic circulation. Two major antiangiogenic factors associated with preeclampsia are soluble fms-like tyrosine kinase-1 and soluble endoglin, which are both increased in preeclampsia. Meanwhile, the level of proangiogenic placental growth factor is decreased. This causes an imbalance in pro- and antiangiogenic factors (3, 6, 8). The release of antiangiogenic and inflammatory factors induces systemic maternal endothelial dysfunction with decreased production of vasodilators as prostacyclin and nitric oxide, increased release of vasoconstrictors as thromboxane, and vascular inflammation. This leads to hypertension, maternal organ dysfunction, and the clinical presentation of preeclampsia (3, 7–9, 15).

Inflammation, endothelial dysfunction, hypertension, and kidney disease are common conditions in non-pregnant women with diabetes of reproductive age. Pregnant women with type 1 diabetes who develop preeclampsia are characterized by impaired vasodilatory capacity and elevated markers of endothelial dysfunction, vascular cell adhesion molecule-1, and intracellular adhesion molecule-1 already in early pregnancy (36). Likewise, the vasoactive markers Atrial Natriuretic Peptide and prorenin are elevated in both early and late pregnancy in women with type 1 diabetes who develop preeclampsia compared with that in women who do not develop preeclampsia (37, 38) Pre-pregnancy vascular dysfunction and systemic maternal endothelial dysfunction thus seem to render women with diabetes more susceptible to developing preeclampsia, even in case of normal placentation (3, 6–8, 15, 39, 40).

### 2.2 Risk factors

Well-recognized risk factors for preeclampsia are hypertension, kidney disease, nulliparity, overweight/obesity, advanced maternal age, multiple gestation, assisted reproduction, preeclampsia in a previous pregnancy, and previous stillbirth (3, 7–11). Additional risk factors in women with preexisting diabetes are poor glycemic control, longer diabetes duration, and microvascular complications as diabetic retinopathy and diabetic nephropathy (20, 23).

### 2.3 Complications

In many women, preeclampsia can be almost asymptomatic and slowly progressing, whereas, in some women, it progresses rapidly, with severe complications even including maternal, fetal, and neonatal death. As preeclampsia is a systemic disorder, many organ systems may be affected. Hypertension in the setting of preeclampsia also contributes to the development of maternal organ dysfunction (15). In severe preeclampsia, maternal heart failure, pulmonary edema, acute kidney or liver failure, liver rupture, coagulopathy, or neurological damage including intracranial hemorrhage can be present (2, 3, 6). A feared and serious complication is progression to eclampsia, a condition with maternal tonic-clonic seizures (2, 3, 6, 15). Women who develop preeclampsia are also at increased risk of cardiovascular diseases and chronic renal conditions later in life (3, 6, 8, 9, 40, 41).

Placental dysfunction with impaired placental blood flow may lead to fetal growth restriction (3, 6). The only causal treatment of preeclampsia is termination of pregnancy, and, in consequence, preeclampsia is closely associated with preterm delivery both in the background population and in women with diabetes (2, 42, 43). Being born preterm increases the risk of perinatal morbidity and mortality, as well as long-term complications such as cerebral palsy and cognitive impairment (2, 42, 44).

## 3 Blood pressure

Hypertension in pregnant women has mainly been diagnosed and managed on the basis of office BP measurement, but out-of-office BP measurement as home BP and 24-h ambulatory BP measurements have become more widely used (6, 45). Office BP is an important part of standard pregnancy care, and higher office BP in early pregnancy is associated with an increased risk of preeclampsia (6, 11, 26, 27). Combined preeclampsia screening models estimate the individual woman’s risk for preeclampsia based on maternal risk factors, BP, biomarkers, and uterine artery Doppler flow (46–48). Some international societies endorse the use of combined preeclampsia screening models as an integrated part of the first trimester screening methods for preeclampsia (13, 26). In pregnant women with preexisting diabetes, chronic hypertension (elevated office BP and out-of-office BP present before pregnancy or newly detected before 20 gestational weeks) (**Table 2**) is associated with an increased risk of preeclampsia (20–22, 36, 49–51). Interestingly, even office BP high within the normal range in early pregnancy is also associated with an increased risk of preeclampsia (20).

**Table 2:** Definition of normotension, white coat hypertension, and chronic hypertension.

**Normotension:** Normal office blood pressure and normal out-of-office blood pressure **White coat hypertension:** Elevated office blood pressure but normal out-of-office blood pressure
**Chronic hypertension:** Elevated office blood pressure and elevated out-of-office blood pressure

### 3.1 Out-of-office blood pressure monitoring

Measurement of home BP offers a possibility of multiple BP measurements in the home environment over the course of several days. Home BP is often widely available, and measurements are well tolerated (52). Outside of pregnancy, home BP is superior to office BP for the prediction of cardiovascular outcomes (53–57). Home BP is recommended when diagnosing hypertension, monitoring BP control, and titrating antihypertensive medication (52, 58, 59). Twenty-four-hour ambulatory BP is another out-of-office BP monitoring method that, outside of pregnancy, is regarded as complementary to home BP, each having advantages and disadvantages, but both superior to office BP (52, 58).

Out-of-office BP measurements are recommended by several international societies (13, 26, 34, 60) to detect white coat hypertension, defined as elevated office BP but normal out-of-office BP (52, 58, 59). White coat hypertension has previously been considered a benign condition in non-pregnant persons and has been associated with a lower risk of adverse cardiovascular outcomes compared with chronic hypertension (61). Routine antihypertensive treatment was, therefore, not considered to be indicated. Instead, lifestyle changes and recurrent follow-up with BP measurements are recommended (52, 58, 59, 62, 63). However, there is increasing evidence that white coat hypertension is not a clinically benign condition, as it is associated with an increased risk of cardiovascular disease and development of sustained hypertension compared with normotension in non-pregnant persons (**Table 2**) (61, 64, 65).

### 3.2 White coat hypertension and preeclampsia

In women with preexisting diabetes, white coat hypertension affects 12% of pregnancies. Among women with preexisting diabetes presenting with elevated office BP in early pregnancy, 84% are being identified as having white coat hypertension (66). Elevated office BP detected later in pregnancy should, therefore, be supplemented with home BP to discriminate between white coat hypertension and development of hypertensive disorders including preeclampsia (66).

**Figure 1:**
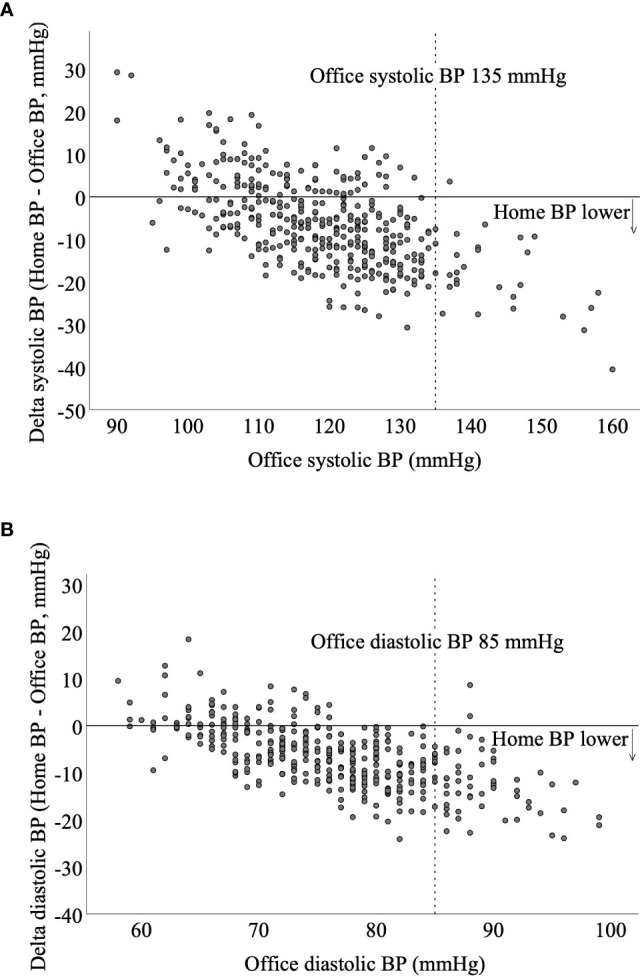
Scatterplots of the difference in home blood pressure and office blood pressure in relation to the office blood pressure in early pregnancy of 404 women with preexisting diabetes. With increasing office blood pressure, the difference between home blood pressure and office blood pressure is greater. **(A)** In women with a systolic office blood pressure equal to or above treatment target of 135 mmHg in early pregnancy, systolic home blood pressure is 19 mmHg lower than office blood pressure. **(B)** In women with office diastolic blood pressure equal to or above treatment target of 85 mmHg in early pregnancy, diastolic home blood pressure is 13 mmHg lower than office blood pressure. Do NC, Home Blood Pressure for the Prediction of Preeclampsia in Women With Preexisting Diabetes, Journal of Clinical Endocrinology & Metabolism, 2022, 18 August 107, e3670–e3678 by permission of Oxford University Press.

In a meta-analysis including almost 5,000 women without diabetes, women with white coat hypertension had a significantly increased risk of developing preeclampsia compared with normotensive women (67). The association between white coat hypertension and preeclampsia in women with pre-existing diabetes has only been sparsely investigated. Recently, a cohort study of 404 women with preexisting diabetes showed that preeclampsia developed more often in women who had white coat hypertension compared with women who had normal BP in early pregnancy (23% versus 7%, p = 0.007) (51). Notably, there were marked differences between home BP and office BP; in early and late pregnancy, home BP was lower than office BP (systolic and diastolic). With increasing office BP, the difference between home BP and office BP was greater. In women with a systolic office BP equal to or above treatment target of 135 mmHg in early pregnancy, systolic home BP was 19 mmHg lower than systolic office BP. In women with diastolic office BP equal to or above treatment target of 85 mmHg in early pregnancy, diastolic home BP was 13 mmHg lower than diastolic office BP (51) (**Figure 1**). This indicates that clinicians should be aware of clinically relevant lower values of home BP in comparison with that of office BP, especially in women with increased office BP.

The evidence on targets for home BP and office BP during pregnancy is conflicting (68–70). Therefore, it has been discussed whether home BP targets during pregnancy should be lower than office BP targets, similar to what is recommended in non-pregnant persons with elevated BP (58, 59, 62). In a recent review and meta-analysis in a diverse group of pregnant mainly normotensive women, both systolic home BP and diastolic home BP were lower than office BP (69). In another systematic review and meta-analysis, no difference in home BP and office BP was seen in pregnant women (70). However, in sub-analyses of women with hypertension, large differences between home BP and office BP were found, whereas little or no difference was seen between home BP and office BP in normotensive pregnant women (70).

In pregnancy antihypertensive treatment of chronic hypertension, targeting a BP of <140/90 mmHg is associated with better pregnancy outcomes, including a lower prevalence of preeclampsia (14). Outside of pregnancy an office BP of 140/90 mmHg is regarded to correspond to a home BP value of 135/85 mmHg (58, 59, 62). In addition, a greater difference in office BP and home BP is seen with higher office BP values, and an office BP of 160/100 is recommended to correspond to a home BP value of 145/90 mmHg in some guidelines (59, 62). No difference is recommended for office BP within the normotensive range (59, 62).

Currently, it is not recommended to treat white coat hypertension with antihypertensive medication in pregnant women nor in non-pregnant persons (13, 34, 52, 58, 60, 68, 71). Nonetheless, with up to 23% of women with white coat hypertension in early pregnancy developing preeclampsia, the current strategy of leaving white coat hypertension untreated in pregnant women with preexisting diabetes is debatable (51).

### 3.3 Blood pressure monitoring and preeclampsia

**Figure 2:**
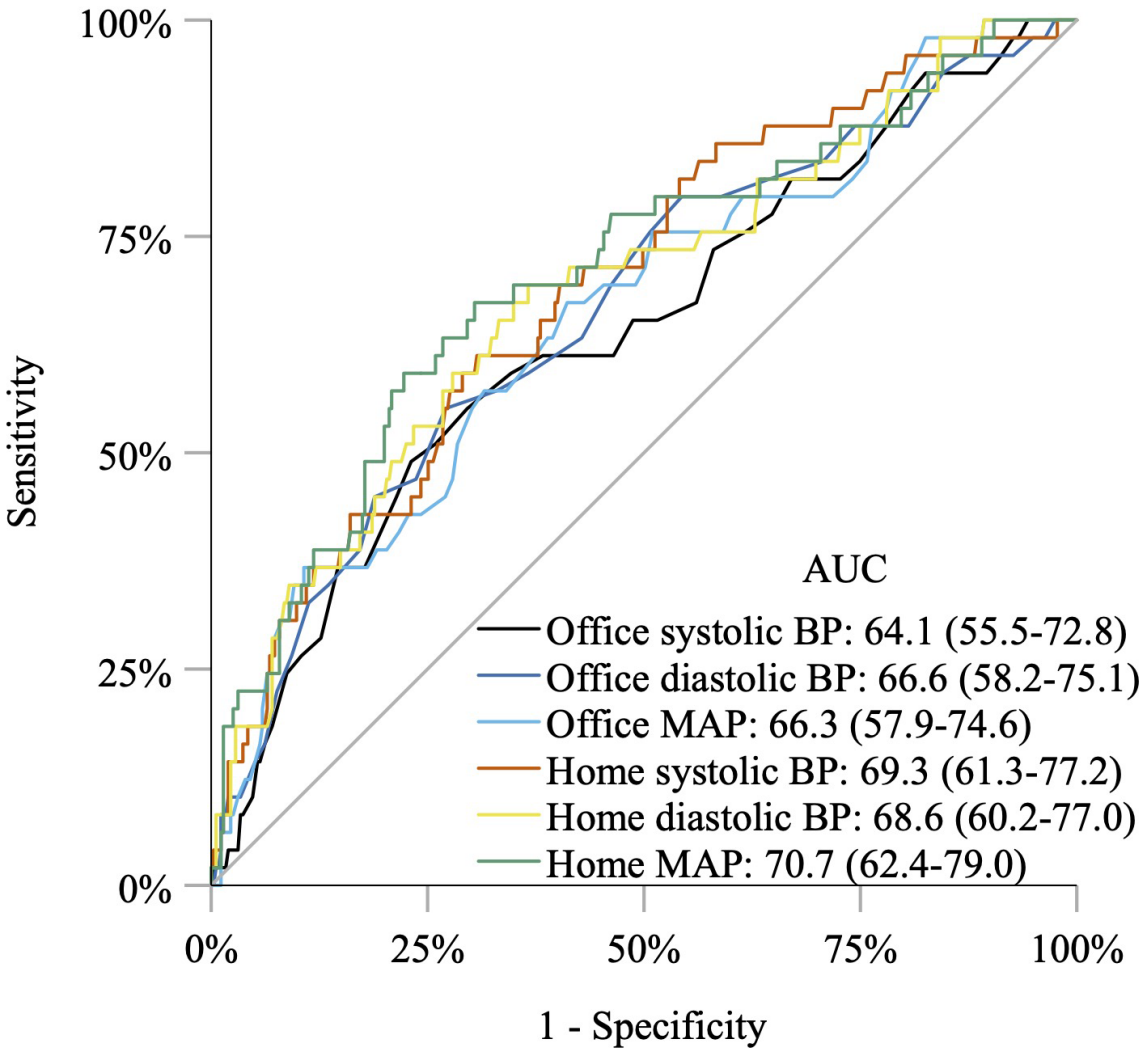
Receiver operating characteristic curves and area under the curves showing that home blood pressure, office blood pressure, and mean arterial pressure are comparable in the prediction of preeclampsia in women with preexisting diabetes. BP, blood pressure; MAP, mean arterial pressure. Do NC, Home Blood Pressure for the Prediction of Preeclampsia in Women With Preexisting Diabetes, Journal of Clinical Endocrinology & Metabolism, 2022, 18 August 107, e3670–e3678 by permission of Oxford University Press.

**Figure 3:**
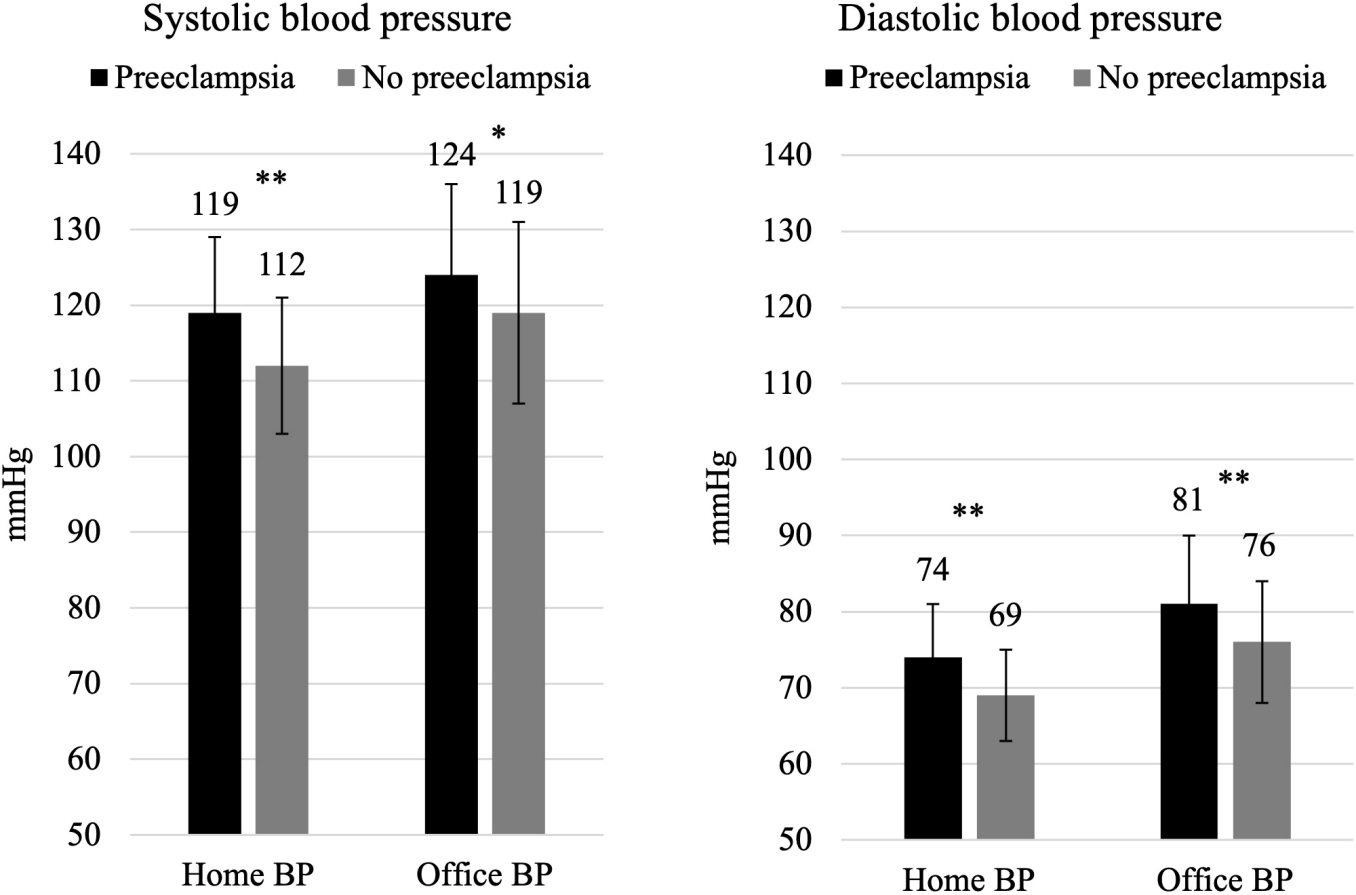
Home blood pressure and office blood pressure values in early pregnancy in 404 pregnant women with preexisting diabetes. Both home blood pressure and office blood pressure are significantly higher in early pregnancy in women who later develop preeclampsia compared with women who do not develop preeclampsia. Data are reported as mean ± SD (error bars). *p = 0.001 and **p < 0.0001.

Both home BP and office BP in early pregnancy are positively associated with the development of preeclampsia. Home BP and office BP are comparable in the prediction of preeclampsia in women with preexisting diabetes, even after adjusting for parity, HbA1c, and diabetic microangiopathy (51) (**Figure 2**). Both home BP and office BP are significantly higher in early pregnancy in women who later develop preeclampsia compared with that in women who do not (51) (**Figure 3**). This is in line with studies in pregnant women with diabetes, where 24-h ambulatory BP was higher in early pregnancy in women developing preeclampsia compared with that in women who did not develop preeclampsia (72–76). In women without a history of hypertension before pregnancy, a higher home BP during pregnancy has also been seen in women subsequently developing preeclampsia (77).

## 4 Physical activity and sedentary behavior

Physical activity is an essential part of the nonpharmacological intervention for hypertension and prevention of cardiovascular disease in non-pregnant persons with and without diabetes (58, 62, 63, 78). Not only is physical activity recommended, but, in recent years, there has been an increasing focus on limiting sedentary behavior, defined as activities with very low energy expenditure such as watching television or reading while sitting (63, 78). There is increasing evidence in non-pregnant persons that sedentary behavior is positively associated with a higher risk of cardiovascular disease and mortality, independent of physical activity (79–83). The National Institute of Health and Care Excellence in the United Kingdom recommends limiting sedentary behavior during pregnancy (84).

During pregnancy, daily physical activity is recommended to both women with and without diabetes because of its potential benefits and low risk of adverse effects (13, 60, 84–86). Physical activity might contribute to lowering the risk of hypertensive disorders including preeclampsia *via* improved endothelial function, placental growth and vascularity, reduced oxidative stress, and lower arterial stiffness (87–89). However, the evidence is not consistent (29, 30, 90).

Sedentary behavior is associated with higher BP, increased inflammation, and decreased insulin sensitivity. These conditions are associated with endothelial dysfunction and oxidative stress (83, 91). Endothelial dysfunction is an important factor in the development of preeclampsia and might also play a role in the association between cardiovascular disease and sedentary behavior (83, 91). In women with preexisting diabetes, endothelial dysfunction is common, and signs of vascular dysfunction in early pregnancy have been demonstrated to precede development of preeclampsia (36).

### 4.1 Physical activity and preeclampsia

In a cohort study of women with preexisting diabetes (92), physical activity and sedentary behavior during pregnancy were assessed by the Pregnancy Physical Activity Questionnaire that is validated for use in pregnancy (93). Sedentary behavior was higher in early pregnancy in women developing preeclampsia compared with the remaining women, whereas total physical activity was similar. Sedentary behavior in early pregnancy was associated with preeclampsia, and, after adjustment for parity, diastolic BP, and smoking, the odds ratio did not change substantially, but the association was no longer significant (92). No other studies on physical activity or sedentary behavior during pregnancy in women with preexisting diabetes (94–97) have examined the possible association with preeclampsia.

## 5 Prophylactic aspirin for prevention of preeclampsia

Aspirin is considered safe during pregnancy, and prophylactic aspirin has, for years, been prescribed to pregnant women at increased risk of developing preeclampsia (98). A beneficial preventive effect of aspirin in women at high risk of preeclampsia has been demonstrated in reviews and meta-analyses of several large randomized controlled trials (RCTs) (35, 98–100).

The underlying mechanism of the beneficial effect of aspirin in pregnancy is still unclear, but different hypotheses have been suggested (98, 99, 101, 102). Aspirin is an anti-platelet agent that, in even low doses (<300 mg/day), inhibits cyclooxygenase 1 activity, leading to a decreased production of the prothrombotic vasoconstrictor thromboxane A_2_ (99, 103). The hypothesized mechanisms are that the inhibitory effect of aspirin on thromboxane improves placentation and reduces placental infarction (98, 99, 101, 102). An imbalance in prostacyclin (vasodilator) and thromboxane (vasoconstrictor) has been demonstrated in preeclampsia and is another mechanism, whereby aspirin might have a beneficial effect to reduce development of preeclampsia (98, 99, 102). The effect of aspirin on preeclampsia seems to be both time and dose dependent. Aspirin should preferably be initiated in early pregnancy before 16 gestational weeks (35, 99, 101, 104) based on the hypothesis that prophylactic aspirin has its primary effect on placentation in early pregnancy *via* improved placentation and reduced placental infarction, thus reducing the placental dysfunction often seen in preeclampsia (98, 99, 101, 102). However, aspirin might also have a beneficial effect if initiated after 16 gestational weeks (105).

Although prophylactic aspirin in low doses is considered safe for use in pregnancy, its use might increase the risk of bleeding, e.g., epistaxis, small skin bleedings, gastrointestinal discomfort, or postpartum hemorrhage (6, 98, 106, 107). A few older studies have raised concern for congenital anomalies including gastroschisis and cryptorchidism after fetal exposure to aspirin in analgetic doses in early pregnancy (108–110), but this has not been found in a recent meta-analysis (111). Prophylactic low-dose aspirin is mainly initiated after the organogenesis and is discontinued around 36–37 gestational weeks, i.e., well before labor normally starts.

In non-pregnant persons with diabetic retinopathy who received aspirin 650 mg/day in the Early Treatment of Diabetic Retinopathy Study, a clinical study of 2,244 eyes that were followed for at least 4 years, aspirin did not increase the risk of vitreous hemorrhage. The authors concluded that there were no ocular contraindications to aspirin treatment when required for cardiovascular disease (112).

At our center, the development of sight-threatening retinopathy during pregnancy in women with preexisting diabetes has remained low in the past two decades (113–115), with no deterioration after implementation of aspirin to all women with preexisting retinopathy (113).

### 5.1 Use of aspirin in women with diabetes

Only few RCTs have recruited and presented results specifically for women with preexisting diabetes. Most of these RCTs used an aspirin dose of 60–100 mg/day, included women beyond 16 gestational weeks, and could not document a favorable effect of prophylactic aspirin for the prevention of preeclampsia (116–119). A systematic review with a meta-analysis of data from RCTs showed no significant difference in preeclampsia between women randomized to aspirin or placebo. However, data within the meta-analysis included relatively few women with preexisting diabetes (120). The largest RCT reporting data on more than 2,500 women, of which 471 had preexisting diabetes, did not demonstrate a beneficial effect of 60 mg/day of prophylactic aspirin initiated between 13 and 26 gestational weeks (117) (**Table 3**). Meanwhile, a published conference abstract of a secondary analysis of this RCT (117) showed a beneficial effect of 60 mg/day aspirin initiated between 13 and 26 gestational weeks in the subgroup of women with preexisting diabetes and hypertension (121).

**Table 3:** Randomized controlled trials comparing aspirin versus placebo during pregnancy, including women with preexisting diabetes.

	Number of randomized Women in total	Number of women with Diabetes	Dose of aspirin	Gestational week at Initiation of aspirin	Preeclampsia in women with diabetes	
					Aspirin	Placebo/ control
ECPPA Collaborative (118), 1996	1,009	62	60 mg	12–32	0%	8.3%
Caritis et al., 1998 (117)	2,539	471	60 mg	13–26	18%	22%
Moore et al., 2015 (116); Secondary analysis of (117)	523	191	60 mg	13–26	18.1%	21.7%
Lin et al., 2021 (119)	990	218	100 mg	12–20	13.0%	11.8%
Rolnik et al., 2017 (122)	1,776	25	150 mg	10–14	No subgroup analysis	

Results for preeclamspia in women with diabetes were reported in four of the trials.

None of the comparisons (aspirin versus placebo) with regard to preeclampsia in women with diabetes were statistically significant.

ECPPA, Estudo Colaborativo para Prevenção da Pré-eclampsia com Aspirina.

In 2017, the Combined Multimarker Screening and Randomized Patient Treatment with Aspirin for Evidence-Based Preeclampsia Prevention (ASPRE) trial comparing aspirin to placebo in 1,776 pregnant women at increased risk of preterm preeclampsia (delivery before 37 gestational weeks with preeclampsia) was published (122). Women were included in the study if they had an increased risk of preterm preeclampsia based on an algorithm combining maternal risk factors including diabetes, mean arterial pressure, uterine-artery pulsatility index, and two maternal biomarkers (122). The study demonstrated a 60% lower incidence of preterm preeclampsia with aspirin versus placebo (1.6% versus 4.3%). Aspirin (or placebo) was initiated between 11 and 14 gestational weeks at a dose of 150 mg/day. However, only 25 women with preexisting diabetes were included in the study, and no sub-analyses were performed in this specific high risk group (122, 123) (**Table 3**).

Previously, diabetes per se did not justify prescription of aspirin. However, in January 2018, shortly after the publication of the results of the ASPRE trial, the American Diabetes Association changed their recommendations to include recommendation of prophylactic aspirin 60–150 mg/day [current recommendation is 100–150 mg/day (19)] to all pregnant women with preexisting diabetes starting at 12 to 16 gestational weeks (19, 124). Similar changes in recommendations have subsequently been made by other international societies (13, 31, 125).

In a prospective cohort study of women with well-controlled preexisting diabetes, the prevalence of preeclampsia was compared in 207 women who were routinely given prophylactic aspirin of 150 mg/day starting in early pregnancy and in 203 women who were only given prophylactic aspirin in case of risk factors for preeclampsia (previous preeclampsia, chronic hypertension, nephropathy, or oocyte donation). The prevalence of preeclampsia was similar (12% versus 11%) in these two cohorts (24).

In two retrospective studies including 716 and 164 women with diabetes, respectively, the prevalence of preeclampsia was compared between women prescribed aspirin before 16 gestational weeks (based on a high risk of developing preeclampsia) and women who were not prescribed aspirin (based on low risk) (126, 127), but this design does not allow firm conclusions.

Overall, the evidence from RCTs of a beneficial effect of routine prophylactic aspirin to prevent preeclampsia in women with preexisting diabetes is lacking (116–120), and a convincing effect has not been shown in real world cohort studies (24, 102, 121, 126, 127).

### 5.2 Pre-pregnancy susceptibility to preeclampsia

In recent years, there has been an increased focus on the maternal cardiovascular system as part of the pathogenesis of preeclampsia, and preeclampsia is no longer only being considered a placental disorder (3, 6–8, 15, 40). Systemic endothelial dysfunction is common in diabetes, and the signs of maternal vascular dysfunction are present already in early pregnancy in women subsequently developing preeclampsia (36). We speculate that this preexisting maternal cardiovascular dysfunction may be exacerbated by pregnancy and plays a pivotal role in the increased risk of preeclampsia, which may contribute to the limited evidence of effect of aspirin in women with preexisting diabetes (24, 102, 116–120, 126, 127).

The prevalence of preeclampsia was similar before and after a change in aspirin prophylaxis strategy both in women with chronic hypertension (128) and preexisting diabetes (24). A secondary analysis of the ASPRE trial examining the effect of aspirin in 110 pregnant women with chronic hypertension showed similar rates of preeclampsia in the aspirin and the placebo group (123). Similar to women with diabetes (36), women with chronic hypertension are often characterized by preexisting endothelial dysfunction (123). The authors of the sub-analysis of the ASPRE trial hypothesized that preexisting endothelial dysfunction was exacerbated by pregnancy. This might play an important role in the development of preeclampsia in these women. Preeclampsia may thus develop even in the absence of placental dysfunction, thereby limiting the effect of aspirin (123).

### 5.3 Preterm delivery and fetal growth restriction

Prophylactic aspirin has been shown to reduce the risk of preterm delivery and fetal growth restriction in women without diabetes (98, 100, 101, 129). This beneficial effect has primarily been seen in studies investigating the preventive effect of aspirin on preeclampsia and could be due to a reduced risk of preeclampsia. However, a recent RCT in pregnant women without diabetes comparing aspirin initiated in early pregnancy to placebo with preterm delivery as primary outcome demonstrated a lower rate of preterm delivery with aspirin use, despite the prevalence of preeclampsia being similar in the two groups (130). Meanwhile, a real-world prospective cohort study of 410 women with preexisting diabetes did not demonstrate a reduced prevalence of preterm delivery in women routinely given prophylactic aspirin compared with that in women only given prophylactic aspirin in case of risk factors for preeclampsia (24). However, women with type 1 diabetes routinely given prophylactic aspirin had higher gestational age at delivery and a lower prevalence of early preterm delivery before 34 gestational weeks, compared with women with type 1 diabetes only given prophylactic aspirin if they had risk factors for preeclampsia. However, the numbers were too small for solid conclusions (24).

Poor placentation in early pregnancy leading to placental dysfunction may also cause fetal growth restriction (3, 8, 15). Aspirin has, therefore, been hypothesized to reduce the risk of fetal growth restriction by improving early placentation and placental function (98, 131).

In women with preexisting diabetes, the prevalence of small for gestational age infants was similar, regardless of prophylactic aspirin being given routinely to all women or given only to women at risk of preeclampsia (24). This is in line with two secondary analyses of the same RCT comparing aspirin to placebo (116, 117, 131). Neither of these secondary analyses, or sub-analyses in pregnant women with diabetes, could demonstrate a difference in the rate of small for gestational age infants between women randomized to aspirin or placebo (116, 131).

## 6 Discussion

Despite extensive research within the field of preeclampsia in the last two decades, prediction, prevention, diagnosis, and treatment of this serious pregnancy complication are still difficult, and understanding of the disease is continuously evolving.

Use of home BP in pregnancy has become more common (6) due to virtual and telephone consultations instead of in-hospital visits. International hypertension guidelines mention home BP in pregnancy, but there is limited guidance on its practical use in terms of cutoff values, when to use home BP, and whether home BP should be included in the diagnostic criteria for preeclampsia (13, 52, 60, 132). Nonetheless, home BP may be a valuable tool to enable BP monitoring, timely initiation and adjustments of antihypertensive treatment, and early detection of deteriorating hypertensive disorders between hospital visits both in women with white coat hypertension and in women who are already diagnosed with hypertension or preeclampsia (13, 52, 69, 133–135). Antenatal visits and hospital admissions might also be reduced with use of home BP (135).

Home BP is affordable, widely available, and more practical than 24-h ambulatory BP and can be used repeatedly over longer periods, such as in pregnancy (52, 58, 62). A disadvantage is the lack of nocturnal readings because nocturnal BP might be higher in women with preeclampsia (136, 137). However, the clinical value of nocturnal BP in relation to pregnancy outcomes is unclear (136). Use of 24-h ambulatory BP may be discomforting especially during sleep (52) and may potentially aggravate the sleep problems that are often seen in pregnancy. Recently, the International Society for the Study of Hypertension in Pregnancy recommended that home BP should be first choice for out-of-office BP monitoring in pregnant women (68). When using home BP, it is important to use a validated device and an appropriately sized cuff (51, 66).

Future studies should investigate, preferably in RCTs, whether pregnant women with white coat hypertension, both with and without diabetes, might benefit from antihypertensive treatment to reduce the risk of preeclampsia. In the meantime, the use of home BP should be considered in case of high office BPs and in women offered telephone consultations.

The Preeclampsia Screening in Denmark (PRESIDE), screening for preeclampsia in the first trimester of pregnancy study (138), is currently investigating a combined screening model for preeclampsia in an unselected Danish pregnant population. If such a combined screening model is found to be clinically valuable, then it would be of interest to validate it specifically in pregnant women with preexisting diabetes, where additional risk factors for preeclampsia are present, and potential biomarkers might differ from healthy pregnant women (23, 139).

Recommendations on being physically active daily during pregnancy are an important part of pregnancy care and are included in current recommendations for pregnant women with and without diabetes (13, 60, 84–86). Likewise, it might be advisable to recommend pregnant women to be less sedentary. There is a need for more research on the role of physical activity and sedentary behavior on the development of preeclampsia in women with preexisting diabetes, ideally as an RCT with physical activity intervention stratified by physical activity level (sedentary or not sedentary).

Despite the limited evidence of prophylactic aspirin for prevention of preeclampsia and other adverse pregnancy outcomes in women with preexisting diabetes (24, 102, 116–120), these women are still recommended prophylactic aspirin of 150 mg/day from early pregnancy (19). Personalized medicine is an important part of the clinical care in women with preexisting diabetes who are dealing with a lot of extra challenges during pregnancy. Although prophylactic aspirin in low doses is considered safe for use in pregnancy and the benefits usually outweigh the potential risks, universal aspirin prophylaxis instead of risk screening in the pregnant background population is not recommended (6). An ongoing, multicenter RCT in pregnant women with preexisting diabetes compares aspirin of 150 mg/day initiated in early pregnancy between 11 and 14 gestational weeks to placebo. The primary outcome is a composite outcome measure of placental dysfunction, including preeclampsia (140). Hopefully, the results of this RCT will shed light on the indications for and effects of prophylactic aspirin in pregnancy in women with preexisting diabetes in the future.

## 7 Conclusions

In the antenatal care of women with preexisting diabetes, screening for and treatment of elevated BP are essential for the prevention of preeclampsia. Home BP and office BP in early pregnancy are positively associated with development of preeclampsia, and home BP and office BP are comparable for the prediction of preeclampsia. However, home BP is lower than office BP, and the difference is greater with increasing office BP. White coat hypertension is not a clinically benign condition but is associated with an elevated risk of developing preeclampsia. Physical activity is associated with a lower risk of preeclampsia in cohort studies. A beneficial preventive effect of initiating low-dose aspirin for all in early pregnancy has not been demonstrated in women with preexisting diabetes.

## Author contributions

ND and LR wrote the first draft of the manuscript. All authors contributed to the concept of the manuscript and read and approved the manuscript. All authors contributed to the article and approved the submitted version.
